# Direct costs of both inpatient and outpatient care for all type cancers: The evidence from Beijing, China

**DOI:** 10.1002/cam4.2184

**Published:** 2019-05-07

**Authors:** Xuejun Yin, Yue Xu, Xiaowei Man, Liming Liu, Yan Jiang, Liying Zhao, Wei Cheng

**Affiliations:** ^1^ The George Institute for Global Health, University of New South Wales Sydney Australia; ^2^ College of Administration Beijing University of Chinese Medicine Beijing China

**Keywords:** cancer, China, cost analysis, healthcare utilization, real world data

## Abstract

**Background:**

Cancer is a major public health issue worldwide. The cost of cancer care imposes a substantial economic burden on society and patient, but it has not been thoroughly studied in China. This study aimed to describe direct cost and cost elements of all cancer types by different beneficial characteristics.

**Methods:**

The research was a retrospective observational study based on inpatient and outpatient records with a primary diagnosis of cancer from 31 hospitals in 2016. Total cost and cost per time were analyzed by cancer type, sources (prescription medicines, consumables fee for diagnosis and surgery, and other health services), and beneficial characteristics (gender and age).

**Results:**

A total of 30 224 eligible inpatient admissions and 485 391 outpatient visits were identified during the study period. Inpatient care costs account for 58.6% cancer treatment costs. Nearly 70% of the total expenditure is spent on patients aged 50‐79 years. Lung cancer had the highest economic cost (15% of overall cancer costs), followed by breast cancer (12%), and colorectal cancer (10%). Anticancer drug cost accounted a large proportion in both inpatient (37.7%) and outpatient care (64.6%). The average cost per inpatient admission was estimated to be $4590.1 (5621.9), ranging from $1157.7 (1349.8) for testis cancer to $7975 (7343.9) for stomach cancer. The regression analyses revealed that length of hospital stay, cancer type, age, payment type, and hospital level were highly correlated with the expenditure per admission (*P* < 0.001).

**Conclusions:**

The cancer care cost is substantial and varies with cancer type. Our findings provide important information for health service planning, allowing more efficient allocation of health resources for the care of people with cancer.

## 1 BACKGROUND

Cancer is the leading cause of death in China as well as worldwide.^1^, ^2^, ^3^ It has become a major public health concern and caused severe economic burdens on society. According to the National Central Cancer Registry of China, there were around 4.3 million new cancer cases and 2.8 million cancer deaths in China in 2015.^4^ The incidence of cancer has increased over the past decades and will continue increasing because of the aging of the population, an increasing prevalence of established risk factors such as smoking, overweight, physical inactivity, and environment changes. The prevalence of cancer survivorship is also rising with the advances in screening, detection, and treatment. Advances in cancer care, including new technologies and targeted therapies, are increasing the case‐specific costs of care.^5^ Researches have shown that disease‐specific expenditure was the highest for cancer.^6^ The catastrophic expenditure on cancer treatment can force patients and households to acute misery. Trends toward greater intensity of healthcare service use and increasing costs of cancer care are expected to result in a greater burden of cancer in the future.

The delivery of affordable cancer care systems requires public health and policy intelligence to incorporate a comprehensive knowledge of the costs of cancer care. Although economic burden of cancer has been assessed in many countries, such as the United States,^7^ the United Kingdom,^8^ India,^9^ and across the European Union,^10^ the current evidence on the cost of cancer treatment in China is limited. So far, studies on cancer in China mostly focus on the incidence and prevalence of cancer, or treatment cost of specific cancer types such as stomach cancer, esophageal cancer, and liver cancer.^11^, ^12^, ^13^ There are few studies providing valuable evidence for the economic burden of all cancer types. The aim, therefore, of this study was to analyze the costs of cancer care for different types of cancers using patient‐level data for a hospital‐based population. Results of the study will provide a detailed baseline, with which to compare future costs and facilitate assessment of the cost‐effectiveness of potential new interventions in cancer control as well as inform an objective public policy framework for the allocation of governmental research funds.

## 2 METHODS

### 2.1 Data source

**Figure 1:**
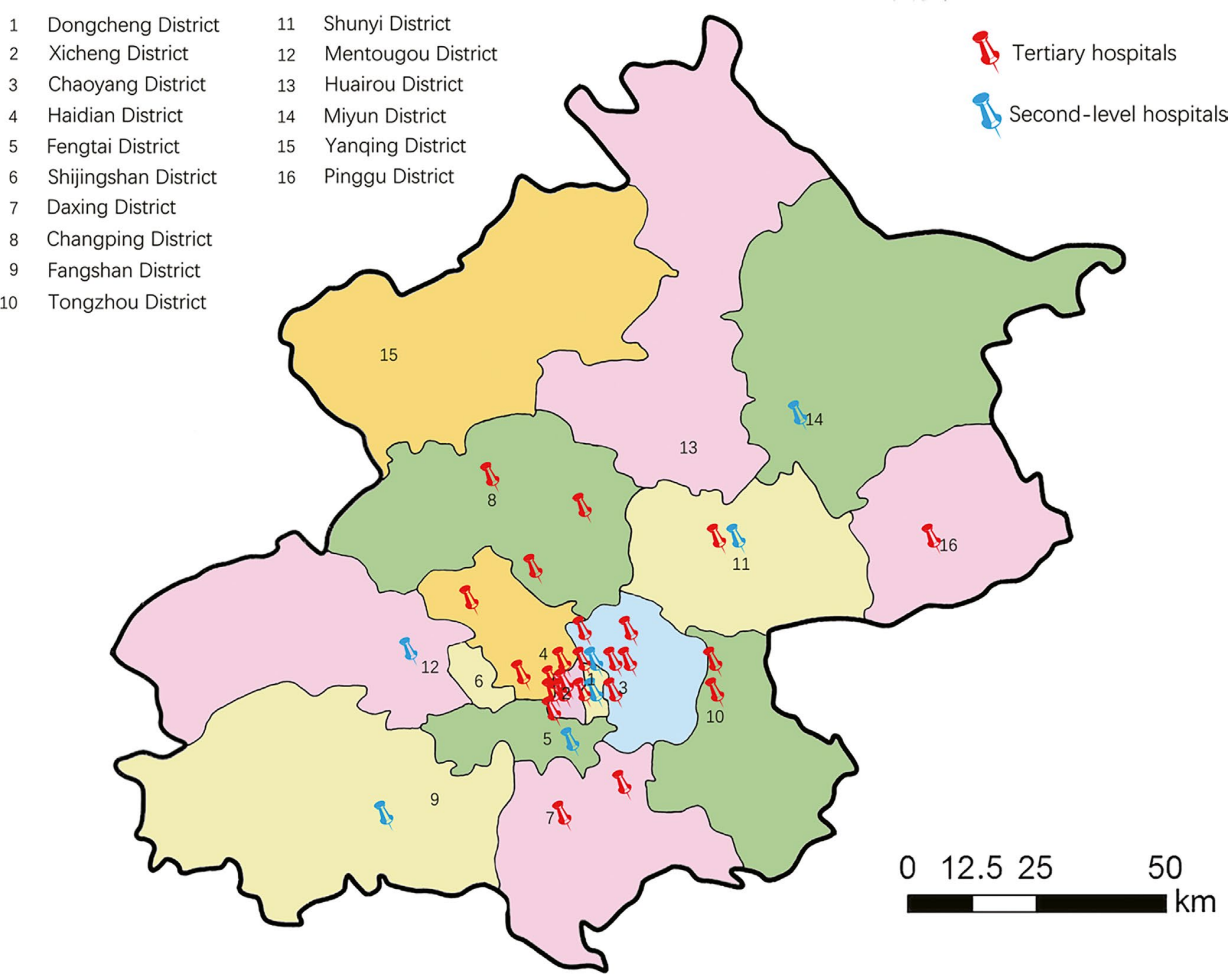
Locations of hospitals included in this study

Patient‐level medical charge records were extracted from 31 general hospitals (24 tertiary level hospitals and seven secondary level hospitals) in Beijing, the capital of China. Tertiary level hospitals are those with over 500 hospital beds providing specialized medical and health services to several regions, and taking teaching and clinical research responsibilities. Secondary‐level hospitals own at least 100 hospital beds providing acute medical and preventative care services to populations of at least 100 000. These hospitals were selected for their health service capacity, location and covering area, and willingness to participate. Figure 1 indicated the locations of hospitals included in this study. The database contains information including sex and age of the patient, date of hospitalization, primary diagnosis, ICD‐10 code of diagnosis, length of stay (LOS), the total cost of hospitalization, and spending from out of pocket. Diagnosis was recorded using the International Classification of Diseases, 10th Revision (ICD‐10). The main reason leading to admission was coded as the primary diagnosis. The patient identification was anonymized, so the ethical committee approval was not required.

### 2.2 Study population

The study population comprised all patients with primary diagnosis as cancer between 1 January 2016 and 31 December 2016. Cancer is defined here by the ICD‐10 codes C00‐97 as the primary diagnosis. Multiple primary cancers were defined using the international rules for multiple primary cancers.^14^ Two well‐trained ICD coders reviewed the name of each diagnosis and its ICD code independently to ensure correct coding. Discrepancies in coding were discussed and then reached consensus. Records missing age, sex, inpatient, or outpatient medical charges were excluded.

### 2.3 Measurement

The primary outcome measures of this study were annual direct medical costs that are associated with services that patients received and are typically measured by specific cancer types, sex, and age. Age was divided into nine 10‐year intervals, except for the last group aged older than 80 years. Medical expenditure consisted of three mutually exclusive types: prescribed medicine, consumables, and hospitalization service expenses. Prescribed medicine cost was split into general medicine fee, traditional Chinese medicine fee, and herb fee. Hospitalization service expenses include hospital care, physician and clinical services, and nursing facility care. Average total expense per visit, cost sources, and varieties in different payment types were analyzed to assess the burden of each hospital admission and outpatient visit. Of China insurance system, Urban Employee Basic Medical Insurance (UEBMI) is created to support employed workers; Urban Resident Basic Medical Insurance (URBMI) is built to support urban residents without a stable job; New Rural Cooperative Medical Insurance (NRCMI) is created to support rural residents. Free medical service provides payments for retired officials (who started work before 1949), civil servant and government‐affiliated employees. Poverty Relief program was designed to cover part of healthcare costs for those in exceptional poverty. Other social insurance included other private commercial insurance companies’ subsidized basic insurance coverage. Expenses of hospital admission and outpatient visit were analyzed separately first, and then summed to get the total annual expenditure. All costs were converted to US dollars ($) based on exchange rates in 2016 (1 USD = 6.6 CNY).

### 2.4 Statistical analysis

Descriptive statistical methods were used to produce a profile of patients’ characteristics, types of cancer, and map the services utilization pattern in terms of proportion of different levels of hospitals and average LOS. Continuous variables were described as mean (SD), and categorical variables were represented by numbers (percentages). We compared hospitalization spending by t‐test and other categorical variables among the group by chi‐square test. The associations of social and medical factors with hospital charges per time are analyzed using multiple linear regression model. Differences of expenditure by varied payment type were examined where generalized linear model was adopted to account for cancer types and patient age. All analyzes were conducted using SAS, version 9.3 (SAS Institute, Cary, North Carolina). A two‐sided *P* value  < 0.05 was considered statistically significant.

## 3 RESULTS

### 3.1 Characteristics of inpatient and outpatient admission

There were 30 224 eligible inpatient admissions with a primary diagnosis of cancer in 2016. Overall, the gender composition was generally balanced (52.8% male vs. 47.2% female). The mean age of the cases was 56.3 (18.0) years. The admission cases aged 50‐69 were more than half of the total cases. The average LOS was 11.4 (9.6) days ranging from 7.5 (6.5) days for Melanoma of the skin cancer to 16.1 (14.1) days for Gallbladder cancer. About half of the cases was supported by the insurance type of UEBMI, URBMI, or NRCMI. Ninety‐four percent of hospital admission was in tertiary hospitals.

A total of 485 391 outpatient visits primarily related to cancer was observed. The mean age of the cases at visit was 54.6 (18.7) years, 59.1% were females, and 96% received diagnosis or treatment in tertiary level hospitals. Similar to age distribution among inpatient cases, significant large proportion of visits among 50‐69 age group was also noted (52.2%). Forty percent of them were supported by the insurance type of UEBMI. Nineteen percent were paying by Free Medical Service, and 17% were self‐paying. Table 1 presents the details of inpatient admissions and outpatient visits.

**Table 1:** Characteristics of cancer inpatient admissions and outpatient visits

	Inpatient admission	Outpatient visit
N	30 224	485 391
Female (N, %)	14 265 (47.2)	286 920 (59.1)
Age, years (Mean, SD)	56.3 (18.0)	54.6 (18.7)
Age group (N, %)		
<10	1324 (4.4)	26 338 (5.4)
10‐19	514 (1.7)	10 328 (2.1)
20‐29	580 (1.9)	10 086 (2.1)
30‐39	1614 (5.3)	29 107 (6.0)
40‐49	3705 (12.3)	65 769 (13.6)
50‐59	7492 (24.8)	126 691 (26.1)
60‐69	8608 (28.5)	126 702 (26.1)
70‐79	4656 (15.4)	64 697 (13.3)
≥80	1731 (5.7)	25 673 (5.3)
Length of Stay, days (mean, SD)	11.4 (9.6)	NA
Payment type (n, %)		
UEBMI	9660 (33.9)	193 482 (40.2)
URBMI	2219 (7.8)	25 058 (5.2)
NRCMI	3485 (12.2)	25 011 (5.2)
Poverty Relief	9 (0.0)	10 (0.0)
Commercial medical insurance	14 (0.1)	6139 (1.3)
Free Medical Service	2392 (8.4)	93 469 (19.4)
Self‐paying	4128 (14.5)	80 148 (16.6)
Other Social Insurance	2028 (7.1)	22 185 (4.6)
Others	4561 (16.0)	36 037 (7.5)
Hospital level (n, %)		
Secondary Hospital	1813 (6.0)	19 082 (3.9)
Tertiary Hospital	28 411 (94.0)	466 309 (96.1)

Abbreviations: NRCMI, New Rural Cooperative Medical Insurance; UEBMI, Urban Employee Basic Medical Insurance; URBMI, Urban Resident Basic Medical Insurance.

### 3.2 Total expenditure by cancer types, gender, and age groups

**Figure 2:**
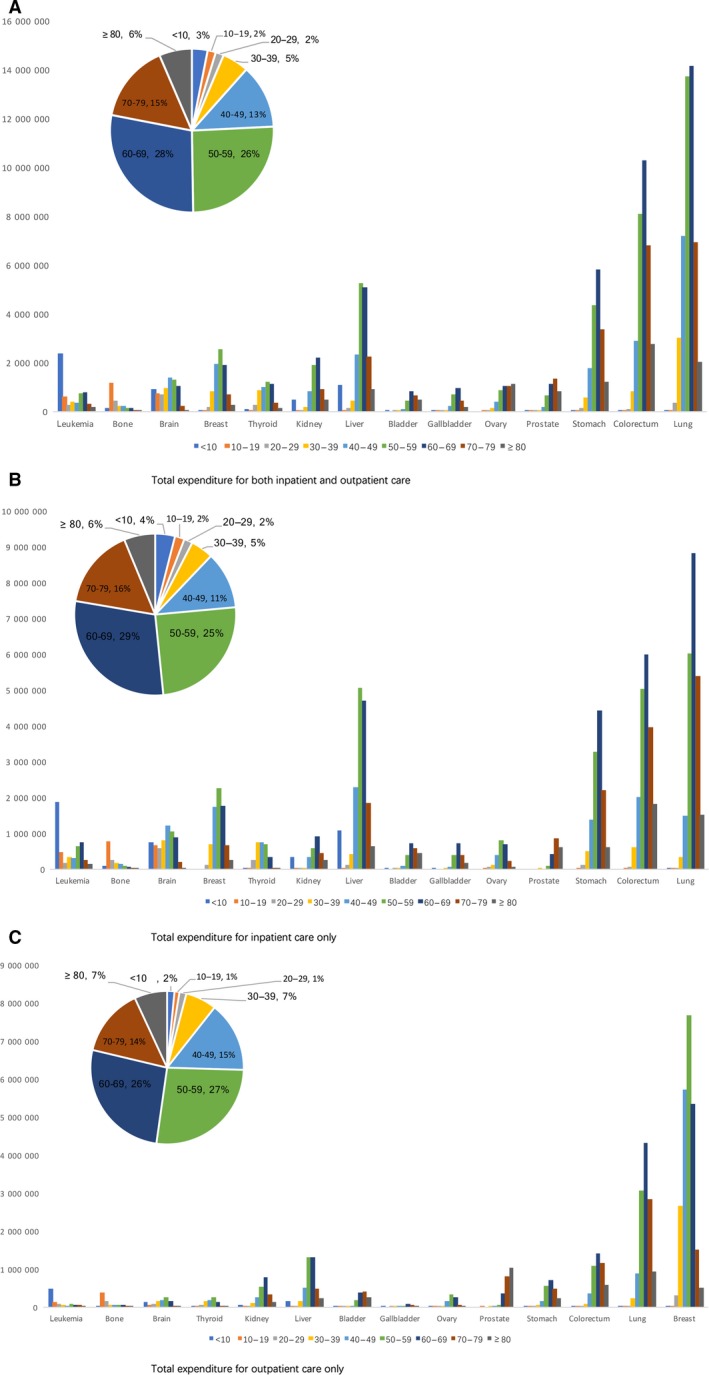
Total expenditure by cancer types and age in 2016. A, Total expenditure for both inpatient and outpatient care; B, Total expenditure for inpatient care only; C, Total expenditure for outpatient care only

Cancer care charged 237.2 million from 31 hospitals in 2016. It was shown that 50.5% was from males and 49.5% from females. Lung cancer had the highest economic cost ($359.2 million, 15% of overall cancer costs), followed by breast cancer ($312.5 million, 12%), and colorectal cancer ($242.3 million, 10%). As for the total inpatient expenditure by age, nearly 70% of the total expenditure was spent on patients aged 50‐79. Higher expenditure was mainly among older groups but leukemia showed that more expenditure happened in children less than 10 years of age. Expenditure on bone cancer was higher in 10‐19 teenagers while spending on brain, breast and thyroid cancer was higher among 40‐59 patients of their working age. Higher expenditure among less than 10 years and older than 50 years of age made the tendency with expenditure of kidney and liver cancer follow a spoon‐shape distribution. (Figure 2).

Inpatient care costs were estimated to be 139.2 million, accounting for 58.6% cancer treatment costs. The highest total expenditure was recorded for lung cancer (23.7 million, 17.0%), followed by colorectal cancer (19.5 million, 14.0%), then by liver cancer (16.2 million, 11.7%), and stomach cancer (12.5 million, 9.0%). These four types of cancer together account for around half of the total inpatient cost of all kinds of cancers. The three highest expenditure cancers among males are lung, liver, and colorectal cancers while among females are lung, colorectal, and breast cancers. Of the total inpatient expenditure, the proportion of costs attributed to prescribed medicines was 37.7%, 28.6% to consumables, and 33.7% to hospitalization service. The proportion of medication expenditure in all hospitalization treatments was lowest for Larynx cancer (25.1%) and highest for lymphomas (48.8%).

**Figure 3:**
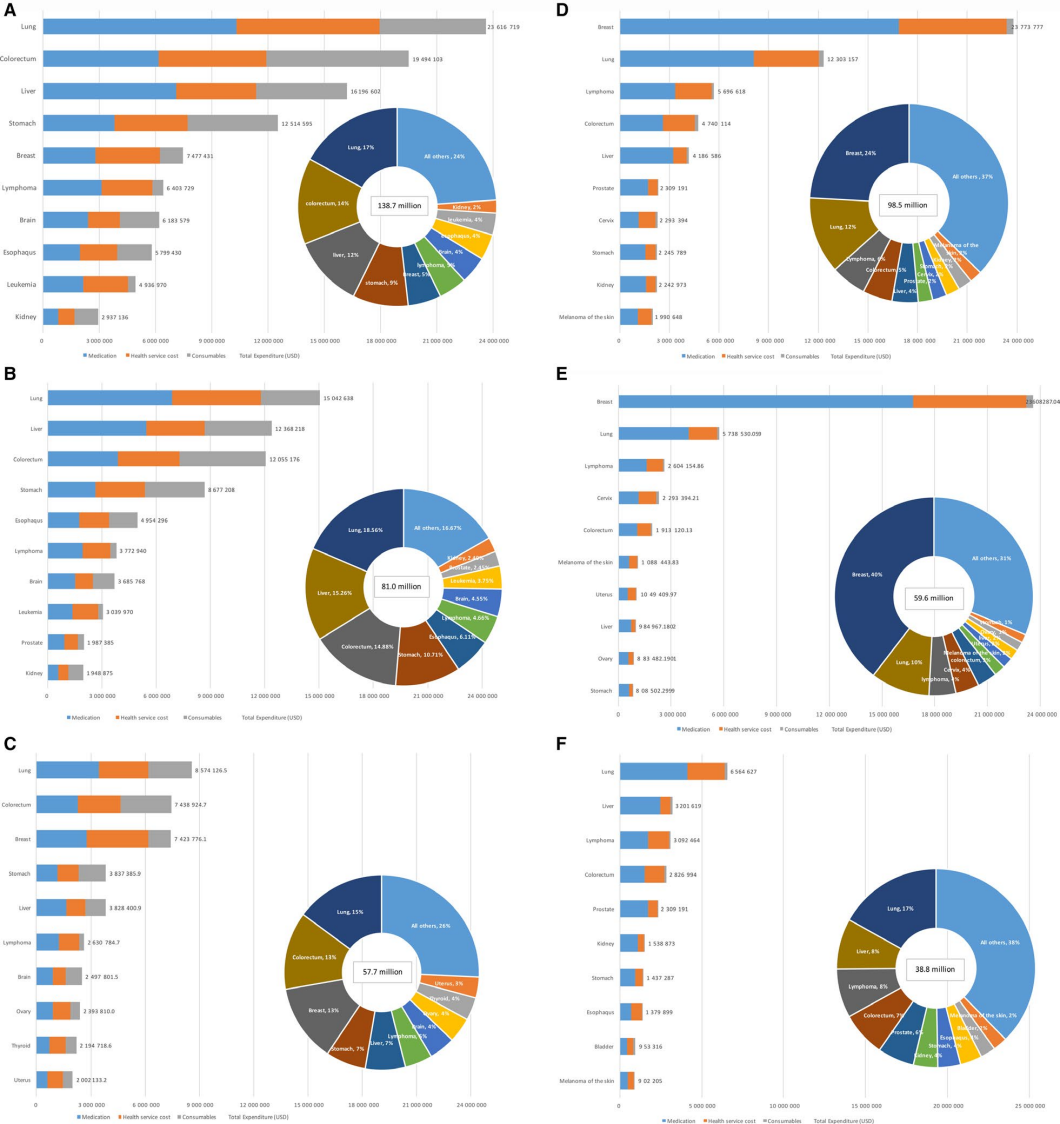
Distribution of costs by cancer type. A, Inpatient costs of 10 most expensive cancers; B, Inpatient costs of 10 most expensive cancers among male; C, Inpatient costs of 10 most expensive cancers among female. D, Outpatient costs of 10 most expensive cancers. E, Outpatient costs of 10 most expensive cancers among male. F, Outpatient costs of 10 most expensive cancers among female

Outpatient care costs were calculated to be 98.5 million, accounting for 41.4% of cancer‐related healthcare costs. Breast cancer incurred the highest outpatient expenditure (23.8 million, 24.1%), followed by lung cancer (12.3 million, 12.5%), and lymphoma (5.7 million, 5.8%). The total expenditure on breast cancer was outstanding among females, accounting for 40% of all the outpatient treatment expenditures. The proportion of costs attributed to prescribed medicines was 64.6%, 2.8% to consumables, and 32.6% to health service. The proportion of medication expenditure in outpatient treatment was lowest for Larynx cancer (28.8%) and highest for liver cancer (77.2%). Figure 3 showed the inpatient and outpatient total expenditure and charges patterns for the 10 highest cancer types by gender.

### 3.3 Charges of each inpatient admission and outpatient visit by cancer types

**Figure 4:**
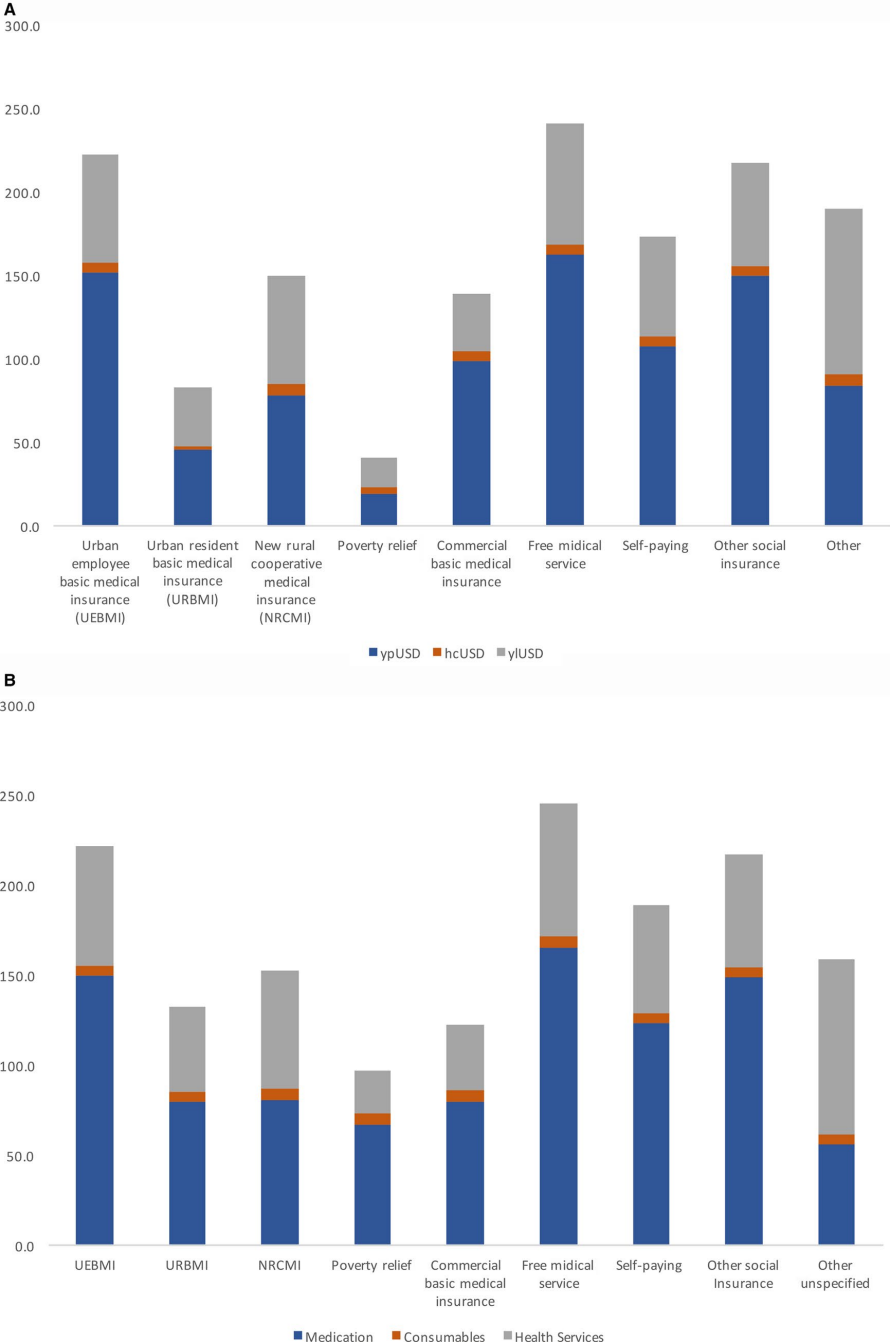
Average expenditure per time by different payment types. A, Average expenditure per inpatient admission by different payment types. B, Average expenditure per outpatient visit by different payment types

Table 2 shows the average expenditure per visit for inpatient and outpatient treatment by cancer types and gender. The average inpatient admission cost was $4590.1 (5621.9) ranging from $1157.7 (1349.8) for testis cancer to $7975 (7343.9) for stomach cancer. The regression analyses revealed that length of hospitalization stay, cancer type, age, payment type, and hospital level were highly correlated with expenditure per admission (*P* < 0.001). The expenditure per in‐hospital permission and the expenditure pattern varied through different payment types. After adjusting age and cancer type, paying by Free Medical Service System was twice higher compared to other insurance payments. Paying by Poverty Relief Program has the lowest mean expenditure. (Figure 4).

**Table 2:** Average expenditure (USD) per case for inpatient and outpatient by cancer types and gender

Cancer types	ICD‐10	Inpatient				Outpatient			
		Male		Female		Male		Female	
		Visits, N (%)	Expenditure per case, Mean (SD)	Visits, N (%)	Expenditure per case, Mean (SD)	Visits, N (%)	Expenditure per case, Mean (SD)	Visits, N (%)	Expenditure per case, Mean (SD)
Lip, oral, cavity & pharynx	C00‐C10, C12‐C14	147 (0.9)	3518.2 (3919.2)	43 (0.3)	3719.7 (2800.1)	1423 (0.7)	173.8 (545.2)	1079 (0.4)	158.3 (420.4)
Nasopharynx	C11	47 (0.3)	2799.5 (2816.7)	14 (0.1)	3766.2 (2642.1)	1103 (0.6)	196.0 (622.6)	432 (0.2)	146.2 (398.5)
Esophagus	C15	656 (4.1)	7552.3 (8188.9)	135 (1.0)	6260.3 (7501.9)	6702 (3.4)	205.9 (512.5)	1404 (0.5)	188.0 (421.0)
Stomach	C16	1107 (6.9)	7838.5 (7312.0)	462 (3.2)	8306.0 (7424.8)	8296 (4.2)	173.3 (332.8)	4080 (1.4)	198.2 (409.7)
Colorectum	C18‐C21	1711 (10.7)	7045.7 (7317.0)	1149 (8.1)	6474.3 (6300.1)	15415 (7.8)	183.4 (453.9)	11295 (3.9)	169.4 (359.5)
Liver	C22	2405 (15.1)	5142.7 (5183.9)	742 (5.2)	5159.6 (5895.6)	12812 (6.5)	249.9 (417.0)	4359 (1.5)	226.0 (372.5)
Gallbladder	C23‐C24	127 (0.8)	6667.8 (7745.5)	106 (0.7)	8691.6 (9675.3)	912 (0.5)	141.5 (293.3)	907 (0.3)	141.5 (275.4)
Pancreas	C25	251 (1.6)	6579.1 (7386.0)	170 (1.2)	6602.0 (7897.0)	1760 (0.9)	147.1 (262.1)	1383 (0.5)	161.8 (311.2)
Larynx	C32	107 (0.7)	3645.2 (3584.3)	5 (0.0)	2453.1 (2498.3)	997 (0.5)	157.1 (529.3)	118 (0.0)	220.2 (775.7)
Lung	C33‐C34	3863 (24.2)	3894.0 (4576.5)	2245 (15.7)	3819.2 (3980.3)	36615 (18.5)	179.3 (373.6)	30057 (10.5)	190.9 (381.7)
Other thoracic organs	C37‐C38	74 (0.5)	5215.5 (5193.7)	44 (0.3)	5513.6 (6315.4)	521 (0.3)	181.3 (535.4)	432 (0.2)	151.3 (274.6)
Bone	C40‐C41	175 (1.1)	5452.6 (6665.5)	139 (1.0)	5237.1 (6990.2)	4076 (2.1)	129.5 (189.7)	3155 (1.1)	119.1 (165.9)
Melanoma of the skin	C43	50 (0.3)	2683.8 (1898.6)	51 (0.4)	3587.6 (2662.6)	4339 (2.2)	207.9 (513.5)	5057 (1.8)	215.2 (502.3)
Breast	C50	25 (0.2)	2146.3 (1631.4)	3364 (23.6)	2206.8 (1794.3)	847 (0.4)	195.4 (250.6)	89028 (31.0)	265.2 (624.1)
Cervix	C53	—	—	563 (4.0)	3511.9 (2944.9)	—	—	12887 (4.5)	178.0 (384.4)
Uterus	C54‐C55	—	—	604 (4.2)	3314.8 (2412.5)	—	—	7019 (2.5)	149.5 (263.7)
Ovary	C56	—	—	788 (5.5)	3037.8 (2984.4)	—	—	7581 (2.6)	116.5 (171.2)
Prostate	C61	679 (4.3)	2926.9 (3656.1)	—	—	8314 (4.2)	277.7 (357.2)		
Testis	C62	18 (0.1)	1577.7 (1349.8)	—	—	143 (0.1)	109.7 (152.8)		
Kidney	C64‐C66, C68	303 (1.9)	6431.9 (5851.2)	166 (1.2)	5953.4 (4940.7)	7029 (3.5)	218.9 (724.5)	4285 (1.5)	164.3 (473.9)
Bladder	C67	493 (3.1)	3583.5 (7745.5)	143 (1.0)	3689.4 (4009.9)	7088 (3.6)	134.5 (247.4)	2606 (0.9)	132.0 (161.5)
Brain, CNS	C70, C72	676 (4.2)	5452.3 (5511.4)	425 (3.0)	5877.2 (6627.2)	3512 (1.8)	190.6 (622.2)	2355 (0.8)	183.9 (576.3)
Thyroid	C73	211 (1.3)	3280.7 (1382.5)	712 (4.9)	3082.5 (1262.2)	2177 (1.1)	93.9 (117.1)	7581 (2.6)	89.3 (101.4)
Lymphoma	C80‐C85, C88, C90, C96	1101 (6.9)	3426.8 (4582.4)	743 (5.2)	3540.8 (5247.5)	18327 (9.2)	168.7 (503.5)	14204 (5.0)	183.3 (530.0)
Leukemia	C91‐C95	454 (2.8)	6696.0 (11432.1)	341 (2.4)	5563.1 (7373.9)	8064 (4.1)	79.9 (238.7)	5409 (1.9)	80.5 (250.8)
All other cancers and unspecified		1279 (8.0)	4654.3 (6403.5)	1111 (8.0)	4661.7 (6579.8)	47999 (24.2)	235.7 (512.5)	70207 (24.5)	210.2 (487.9)

The average outpatient care cost was $202.9 (478.0) per visit ranging from $80.2 (243.6) for leukemia to $277.7 (357.2) for prostate cancer. Age, hospital level, gender, and cancer type were significantly correlated with the expenditure per visit (*P* < 0.001). The mean total outpatient visit expenditure was still the highest in patients paying by Free Medical Service System compared to other payment types. (Figure 4).

## 4 DISCUSSION

Cancer is a major public health concern to the utmost relevance for China because of its large population base, very high treatment costs and poor survival prospects. Cancer is known as the most expensive disease but the distribution and expenditure patterns of cancer care have not been thoroughly studied. To our knowledge, this study is the latest and one of very few studies reporting the costs by cancer type, sources (prescription medicines, consumables fee for diagnosis and surgery, and other health services), beneficial characteristics (gender and age) for both inpatient and outpatient treatments. Results of our study has the potential to support new researches producing much needed evidence to achieve the efficient allocation of current and future health resources.

We found that inpatient hospitalization represents a larger component of spending on cancer similar to studies conducted in other countries.^15^, ^16^ Cancer cost was predominant in people aged from 50 to 70. This supports existing evidence in China and consistent with epidemiological findings that most cancer incidence rates increase exponentially with aging.^17^ As for the expenditure pattern, we found that cost attributed to medication accounted a large proportion in both inpatient (37.7%) and outpatient care (64.6%) across all kinds of cancers. This anticancer drug cost proportion is much higher than that in European Union of 27%.^10^ Half of spending on anticancer medicines in China was generated by imported medicines and a large part of them is out of medical insurance reimbursement scope.^18^ Drug cost imposes huge burden on patients that some are not able to receive continuous treatment or tend to take less treatment.^19^ Although the Chinese Government has exempted tariffs on imported cancer drugs and incorporated more cancer drugs into the catalogue of medical insurance reimbursement, further steps need to be taken to develop affordable and effective medicines for patients in China. It is crucial to invest in drug research and strengthen China's own vibrant and innovative pharmaceutical industry, in collaboration with different stakeholders, particularly academia. Besides, promoting the of price “transparency”, legislation is always encouraged.

We identified the evidence that lung cancer had the highest economic cost (15% of overall cancer costs), followed by breast cancer (12%) and colorectal cancer (10%), which is consistent with previous studies domestic and aboard.^10^, ^20^ The higher total expenditure in lung, colorectal, liver and breast cancer was due to larger number of visits, which hints the disease map in Beijing.^21^ Health policies should aim to curb universal risk factors causing those kinds of high prevalence cancerous tumors, such as tobacco and alcohol, poor diet (insufficient fruit or vegetable intake), overweight and obesity, physical inactivity, chronic infections from Hepatitis B and C virus, and environmental risks. It is also worth noting that breast cancer cost accounted for 40% of all the cancer care spending in female outpatients. High prevalence of breast cancer among reproductive females cautions significant policy attention to reduce increasing burden of breast cancer among females. Studies have proven that screening and an earlier diagnosis can generate substantial savings.^22^, ^23^, ^24^ Priorities need to be put on prevention, early detection, proper diagnosis, and cost‐effective treatment of breast cancer among females.

The average direct inpatient care costs of all types of cancers were estimated as $4588.94; this is much higher than $1671.8 that was found in another study investigating inpatient cancer care burden in Anhui, an inland province of China in 2014.^25^ This may be explained mainly by several reasons. For one thing, our study included more tertiary hospitals that charged higher than secondary and township hospitals.^20^ For another, Beijing is characterized by concentrating high quality medical resources of the whole country and patients with more serious conditions tended to seek treatment in Beijing's hospitals and are intensively treated. Lastly, estimation from “household‐based” data rather than hospital‐based data may be underestimated due to recall bias. The total expenditure is high for lung cancer, liver cancer, and breast cancer due to high prevalence while the average inpatient cost per case was found to be higher for stomach, gallbladder, and esophagus cancer with more than $7300 for each inpatient admission. This may be associated with higher prevalence of surgery or longer in‐hospital stay.

Various insurance policies influenced the health expenditure of patients.^26^, ^27^ UEBMI and URBMI are the mainstream health insurance schemes in urban China. As observed in our study, patients supported by UEBMI will pay more for inpatient treatment than those supported by URBMI. Similar finding has been reported from a study in evaluating the disparity in reimbursements for tuberculosis care among the abovementioned three health insurance schemes.^28^, ^29^ Patients belonging to enrollees of the new corporative medical care and the city resident medical insurance incurred relatively lower per case direct expenditure than patients belonging to other insurance sachems. It may because these two insurance scheme had enacted the stricter policies and audit procedures in refunding medical care expenses and they also have lower imbursement rates than UEBMI.^30^ We identified that patients supported by free medical service spent three folds higher than other insurance payment and patients supported by poverty relief had least expenses, which have never been revealed by previous studies due to their data source limitation. Insurance benefit package was designed to promote efficient service utilization. Special attentions need to be paid to the efficiency of free health service program. On the contrary, patients supported by poverty relief program were found to have lowest total expense per visit. It is possible because the high expenditure for hospital admission became the barrier for the poorer people seeking proper treatment. Although patients under the poverty line enjoyed poverty relief program documented by national policy, they were still faced with relatively heavy financial burden.

There are several limitations to this study. Firstly, the cancer cases may not include all cases in Beijing, but the healthcare costs were obtained from the System of Health Accounts which included the representative samples for estimating provincial total expenditure and was submitted to National Health Organization Center for Health Development. Data were directly extracted from the hospital financial system so that they are most complete and valid than any other cost data sources. Additionally, the distribution of hospital visits due to specific cancer types was reasonably similar to that for all incident and prevalent cancers in Beijing in 2016.^21^ It is reasonable to believe the results are representative for the situation in Beijing. Secondly, economic development, cancer type distribution, and healthcare costs significantly varied in different areas in China. Although the data were representative of cases in Beijing, it may lead to imprecise estimates when weighted to population‐level distributions in the whole country. Thirdly, we restricted our analysis to direct health system costs due to a lack of comprehensive data for other indirect costs. Overall, our estimates are likely to underestimate the total health services costs of cancer care for the reasons detailed above. Fourthly, the database has not recorded the stage of cancer, so we are not able to o estimate incidence and prevalence costs of cancer separately. The costs of cancer care were included no matter if it was initial treatment, end‐of‐life care, or continuing care costs for cancer survivors. Future research with these data could include analyses of indirect costs such as productivity costs, costs borne by carers, and burden of disease. Others have been able to report on costs by disease stage where the data were available; however, detailed breakdown by stage was beyond the scope of this study. Our future work will focus on individual cancer types, with a more detailed description of costs by various patients and tumor characteristics, including disease stage. Further research could also include projections of cancer prevalence by phase of care to estimate future costs.

This study has several other key strengths. Previous studies have described the strengths of using real world data for this type of research and the usefulness of administrative health datasets for costing studies. We used a large hospital‐based database with detailed individual‐level data, including all cancer types, both inpatient and outpatient records to reflect the actual treatment expenditure of cancer cases. The findings presented here accurately reflect the real situation in Beijing, are more inclusive than previously reported estimates, and very important for understanding the influences of healthcare expenditure.

## 5 CONCLUSIONS

With an aging population, increasing prevalence of established risk factors and advances in cancer treatment, the prevalence of cancer in China will continue to grow in the years to come, resulting in a huge burden on patients, society, and health system. Describing the characteristics of cancer treatment spending is an essential first step in elucidating its treatment patterns and producing data‐driven evidence for major stakeholders. The costs of cancer care vary substantially by cancer types and medical insurance types. Medication costs account for the largest proportion of total expenditure. The factors, which drive increased spending and determine whether changes in particular subcategories of spending, have been associated with improvements in processes or outcomes. Results of our research provide important information for health services planning, implementation and delivery, and the evaluation of potential new interventions in cancer control, allowing more efficient allocation of health resources for the care of people with cancer. Our study suggests the key areas for future efforts include developing and enhancing research resources, improving estimates and projections of burden, particularly indirect costs, evaluating use and effectiveness of targeted therapies, and financial burden of cancer for patients and their families.

## DATA AVAILABILITY STATEMENT

The data that support the findings of this study are available from the corresponding author upon reasonable request.
